# Percutaneous closure of a traumatic ventricular septal defect: a case report and literature review

**DOI:** 10.1186/s12245-024-00805-z

**Published:** 2025-01-09

**Authors:** Camilo Andres Calderon-Miranda, Maria Juliana Reyes-Cardona, Gabriel Roberto Lopez-Mora, Fernando Andrés Guerrero-Pinedo, Jairo Sanchez-Blanco, Carlos Enrique Vesga-Reyes, Jorge Alexander Zambrano-Franco, Pastor Olaya

**Affiliations:** 1 Departamento de Cardiología, Fundación Valle del Lili, Carrera 98 No. 18 - 49, Cali, 760032 Colombia; 2 Facultad de Ciencias de la Salud, Universidad Icesi, Calle 18 No. 122-135, Cali, 760031 Colombia; 3 Centro de Investigaciones Clínicas, Fundación Valle del Lili, Carrera 98 No. 18 - 49, Cali, 760032 Colombia; 4 Departamento de Medicina Interna, Fundación Valle del Lili, Carrera 98 No. 18 - 49, Cali, 760032 Colombia; 5 Unidad de Intervencionismo Vascular, Fundación Valle del Lili, Carrera 98 No. 18 - 49, Cali, 760032 Colombia

**Keywords:** Penetrating cardiac trauma, Ventricular septal defect, Emergency thoracotomy, Percutaneous closure, Echocardiography

## Abstract

**Background:**

Penetrating cardiac trauma is an entity with high pre and intrahospital mortality due to complications such as cardiac tamponade and massive hemothorax. A ventricular septal defect (VSD) occurs in 1–5% of cases and can present early or late. The management strategy for VSD resulting from penetrating cardiac trauma is uncertain.

**Case presentation:**

A 19-year-old man was admitted in cardiorespiratory arrest after a precordial stab wound. Cardiopulmonary resuscitation was initiated achieving return of spontaneous circulation. eFAST evaluation revealed cardiac tamponade, he was taken to emergency left thoracotomy finding a perforation of the free wall of the left ventricle and a tear of the upper lobe of the left lung that were sutured. The patient was discharged and six days later was readmitted with fever and dyspnea. During treatment for a surgical site infection a new-onset pansystolic murmur was found: A transthoracic echocardiogram revealed a 13-mm VSD with left-to-right shunt. A multidisciplinary team recommended percutaneous closure of the defect which was successfully performed without complications.

**Conclusions:**

Traumatic VSD is a rare complication of penetrating cardiac trauma. A thorough clinical and echocardiographic evaluation is essential for its diagnosis and characterization. Symptomatic septal defects, those 10 mm or larger, with Qp: Qs greater than 1.5, or causing complications such as pulmonary hypertension or valvular involvement, are usually closed to prevent progression of heart failure. Management of traumatic VSD has traditionally been surgical. However, a percutaneous intervention is a viable alternative in selected stable patients. Unlike ischemic VSD, early intervention after patient stabilization generally yields favorable outcomes.

**Supplementary Information:**

The online version contains supplementary material available at 10.1186/s12245-024-00805-z.

## Background

Penetrating cardiac trauma is an entity with a high prehospital mortality, most frequently seen in young male patients and presenting with complications such as cardiac tamponade and massive hemothorax [1]. Mortality is highest in projectile injuries, reaching 90%, and ranges from 16 to 67% in stab wounds. The spectrum of traumatic cardiac injuries includes pericardial, myocardial, valvular, septal and vascular injuries. Ventricular septal defects (VSD) are the most common traumatic septal injuries and may present early or late [2]. The management of VSD resulting from penetrating cardiac trauma is uncertain, with limited information available on the best approach.

## Case presentation

A previously healthy 19-year-old patient was brought to the emergency room in cardiopulmonary arrest after a precordial stab wound. Cardiopulmonary resuscitation was started and return to spontaneous circulation was achieved; Extended Focused Assessment with Sonography for Trauma (eFAST) showed cardiac tamponade. Emergent left thoracotomy was performed revealing 500 cc hemothorax, hemopericardium, an 8 mm perforation on the anterolateral wall of the left ventricle requiring repair with two pledget-reinforced 3 − 0 polypropylene interrupted sutures; and a perforation of the upper lobe of the left lung with layer bleeding and air leak, which was repaired with 1 polyglactine 910 suture, a chest tube was inserted. The postoperative transthoracic echocardiogram had no evidence of shunts, left ventricular ejection fraction was 60–65%. The patient was discharged after removal of the chest tube without complications.

**Fig. 1:**
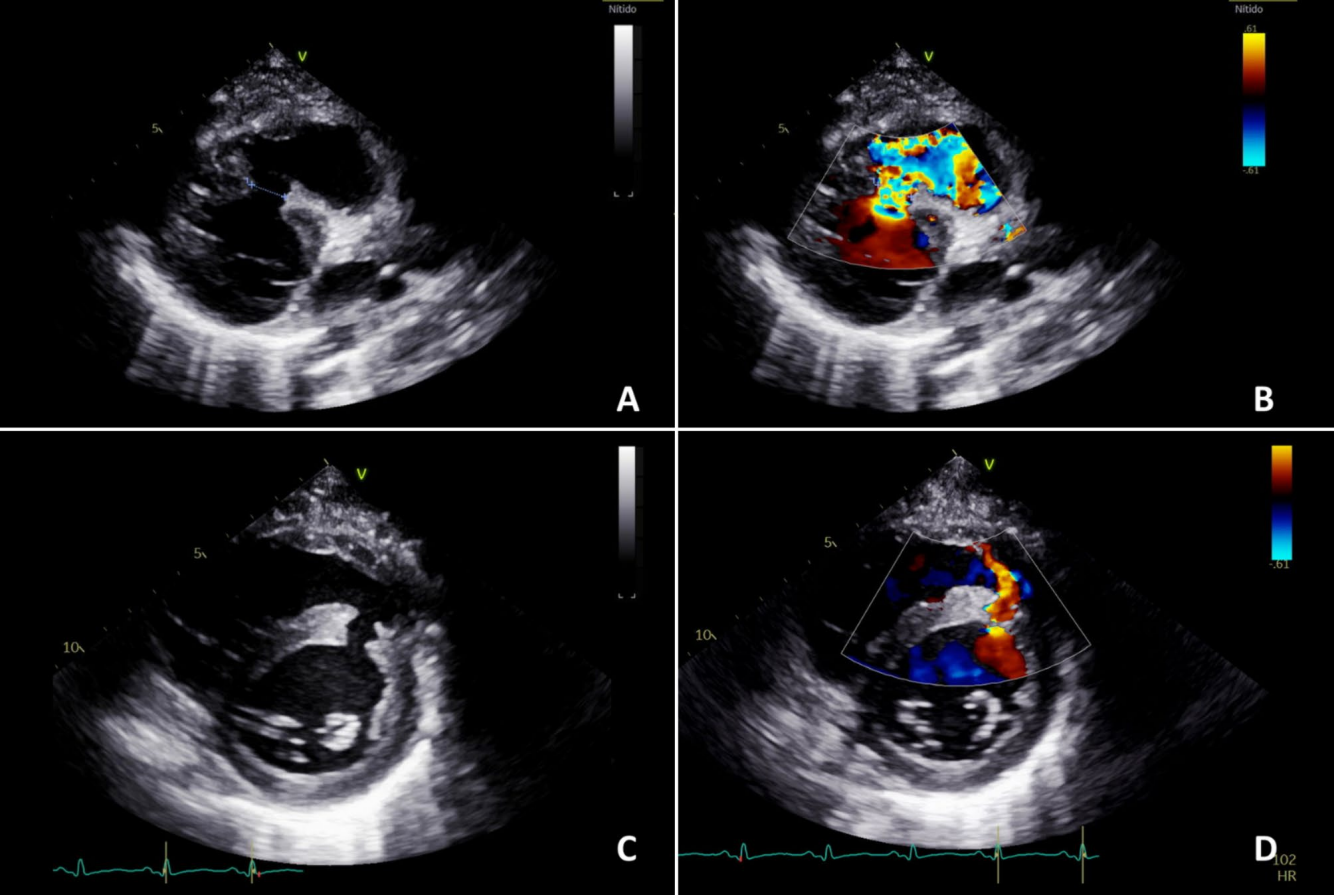
Transthoracic echocardiogram. **A**. Parasternal long-axis view: 13 mm ventricular septal defect (VSD). **B**. Color Doppler: Left-to-right shunt across the VSD **C**. Parasternal short-axis window: VSD **D**. Color Doppler showing flow through the VSD

Six days later, he was readmitted with fever, chills, dyspnea, hemoptysis, seropurulent secretion and local pain in the surgical site. In the initial evaluation he was normotensive, tachycardic, afebrile and had decreased breath sounds in the left lung field. A thorax computed tomography (CT) was performed showing multilobar consolidation in the right lung field and bilateral left-predominant pleural effusion. Left thoracentesis was performed obtaining a transudate and negative cultures. Antibiotic treatment was started with piperacillin/tazobactam and vancomycin. During follow up, the patient persisted with dyspnea and tachycardia, and a new-onset harsh pansystolic heart murmur with thrill was documented. A transthoracic echocardiogram (TTE) revealed a VSD with a maximum diameter of 13 mm and left-to-right shunt, mild dilation of the left chambers with normal systolic function (left ventricular ejection fraction of 63%) and a right ventricle with normal size and contractile function (Fig. 1, see Supplementary file video 1). Previous trauma was considered the causal agent of the VSD.

**Fig. 2:**
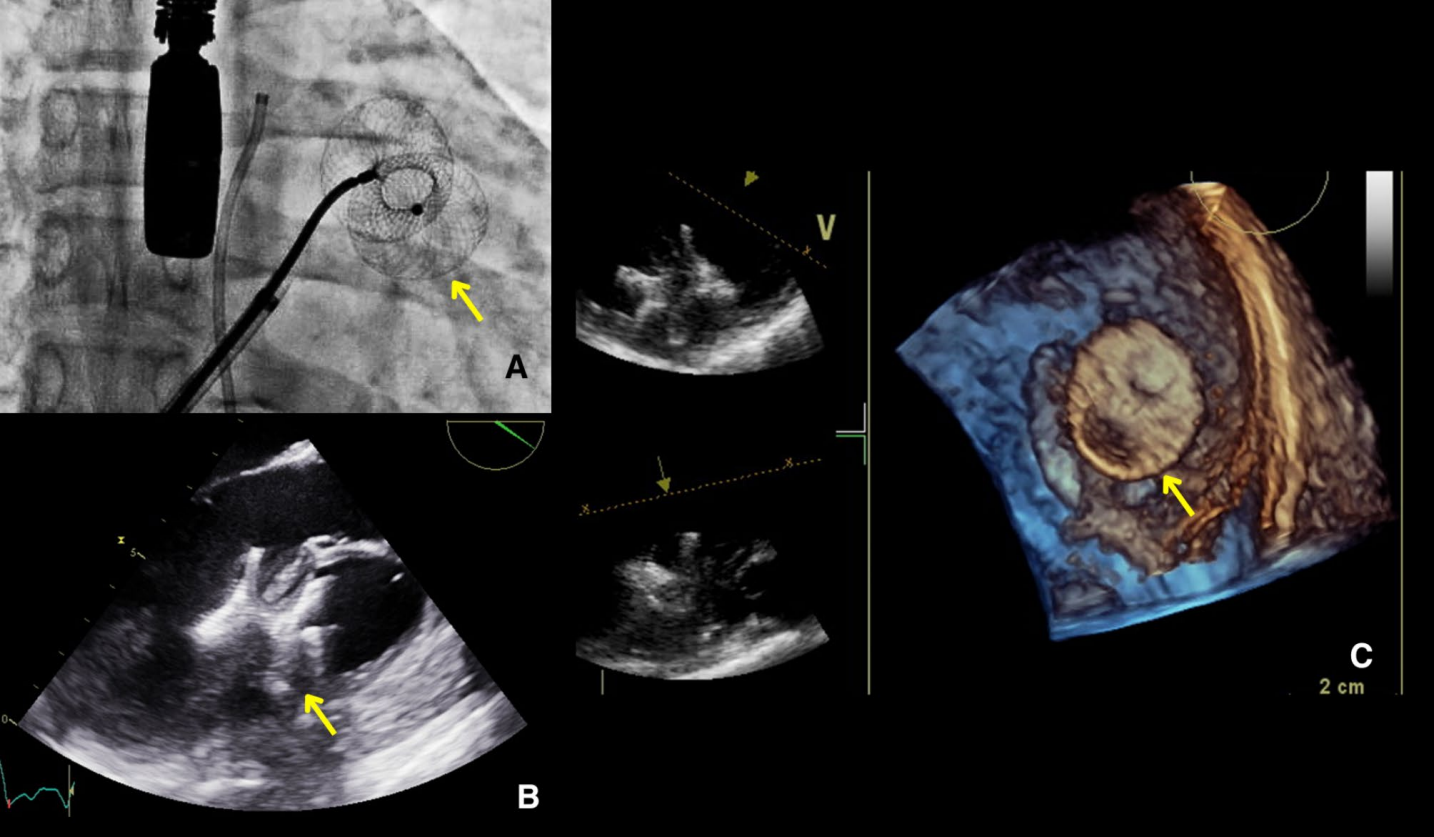
Intraoperative fluoroscopic and transesophageal echocardiogram (TEE) guidance. **A**. Fluoroscopy showing the deployment of an Amplatzer device (yellow arrow). B. TEE Parasternal long-axis window: Amplatzer device well positioned (yellow arrow). **B**. TEE 3D reconstruction of the Amplatzer device (yellow arrow) occluding the VSD

**Fig. 3:**
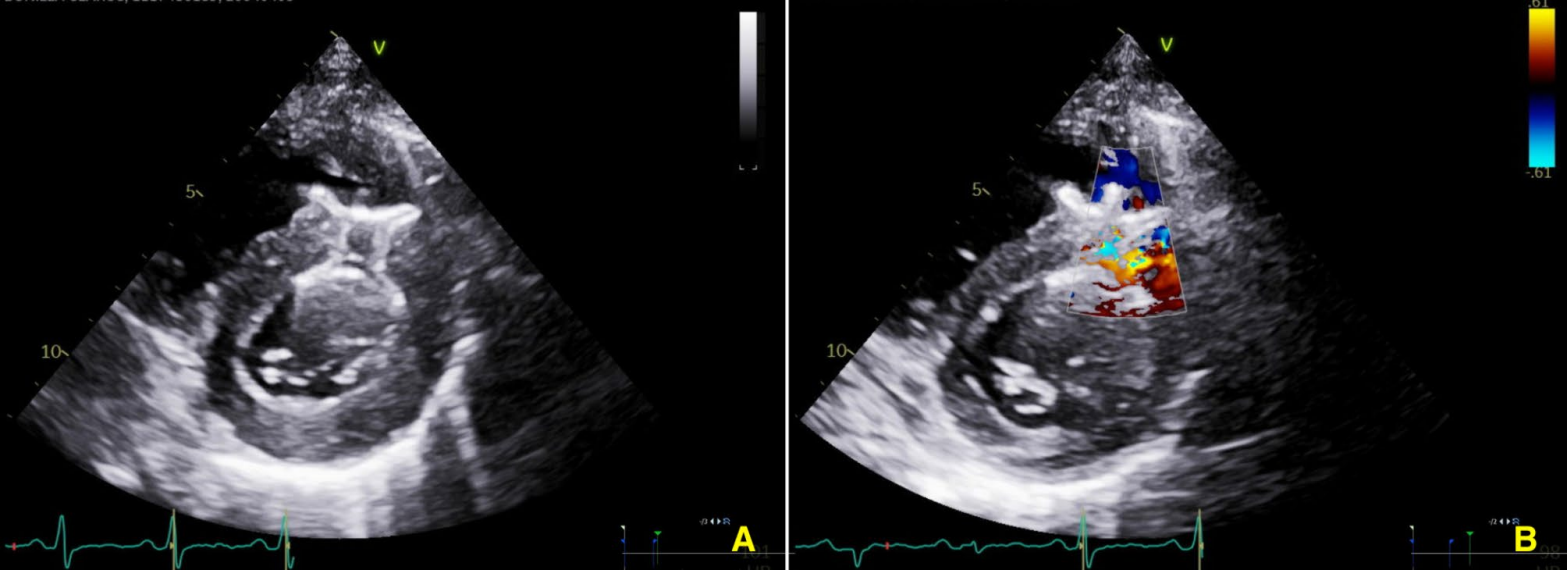
Transthoracic echocardiogram. Parasternal short-axis view **A**. A well-positioned VSD closure device is observed in the middle segment of the anterior septum. **B**. Color Doppler: Unidirectional left-to-right residual shunt, with a large decrease in flow compared to the preprocedural study

A multidisciplinary evaluation by cardiovascular surgery and interventional cardiology took place, deciding to perform a percutaneous closure of the VSD. The procedure was performed using a right femoral arterial and left femoral access approach, with fluoroscopic and transesophageal echocardiogram (TEE) guidance (Fig. 2). TEE showed a muscular VSD measuring 11 mm x 12 mm from the left ventricle and 18 mm x 10 mm from the right ventricle, with a left-to-right shunt with peak velocity 4.2 m/s, estimating a 71 mmHg gradient. A 22 mm Muscular VSD Occluder Amplatzer P.I device was selected according to the sizing and device selection instructions provided by the manufacturer, which advise choosing a device 3 mm larger than the maximum diameter of the defect by echocardiographic measurements. Through right heart catheterization a Pulmonary-to-Systemic Flow Ratio (Qp: Qs) index of 1.93 was calculated, consistent with a shunt with significant hemodynamic repercussions. The defect was crossed retrogradely with an Amplatz Left 2 (AL2) catheter, the Balanced Middle Weight (BMW) 0.014 guidewire was successfully advanced. A 120 cm Argon Atrieve Vascular Snare Kit catheter was advanced through the internal jugular vein and the guidewire was tied in. However, there was not enough support to advance the introducer of the releaser. The defect was crossed again with a hydrophilic exchange guidewire. The guidewire was caught with the loop catheter, externalized and an arterio-venous circuit was made. The device release system (10 Fr) was advanced via the femoral vein and passed antegradely through the defect. The 22 mm Muscular VSD Occluder Amplatzer P.I device was deployed across the defect. After checking its position and stability, its release was carried out. Postoperative transthoracic echocardiogram showed the device was adequately positioned with minimal residual unidirectional flow from left to right (Fig. 3, see Supplementary file video 2). The patient had a favorable clinical course with resolution of the dyspnea and was discharged without complications.

## Discussion and conclusions

This case describes a muscular VSD secondary to penetrating cardiac trauma, successfully repaired with a percutaneous intervention. A traumatic VSD is an uncommon complication of penetrating cardiac trauma, occurring in 1–5% of cases [3]. It may present early caused by direct injury in penetrating trauma or by compression of the heart between the sternum and vertebrae in blunt trauma; or late, caused by edema, ischemia, subsequent necrosis and perforation of the septum after traumatic injury [4]. Clinical presentation of VSD varies from absence of symptoms to shock, and may be insidious or delayed, demanding an exhaustive physical examination including auscultation for a new-onset heart murmur as was documented in our patient. Initial diagnostic assessment includes a 12-lead electrocardiogram, serum troponin levels and a transthoracic echocardiogram. Electrocardiography is normal in more than half of VSD cases, with the most common abnormalities being intraventricular conduction delays including right bundle branch block [5]. Elevated troponin levels after trauma are indicative of myocardial injury and warrant a structural evaluation.

Echocardiography plays an essential role in the diagnosis of VSD. TTE has an outstanding detection rate up to 95% [5], with better sensitivity in defects larger than 5 mm. Echocardiography enables characterization of morphologic features and location of the VSD, measurement of chambers size and function, identification of associated complications such as pulmonary hypertension and color doppler evaluation of the size, timing and direction of the shunt. The magnitude of left-to-right shunting and subsequent hemodynamic significance is most accurately assessed through the pulmonary to systemic flow ratio (Qp: Qs). Due to technical limitations, TTE estimated Qp: Qs has poor correlation with oximetry-derived Qp: Qs measured through cardiac catheterization, which is the gold standard [6]. TEE is reserved for patients with deficient TTE windows and to identify associated valvular abnormalities. 3D echocardiography enables visualization of the defect from different perspectives, adds depth to the rendering and allows for accurate measurement of the defect, which is useful for management planning, especially for the placement of a septal closure device [7].

There is limited literature on the treatment of traumatic VSDs. It depends on the size of the defect, its location, hemodynamic assessment and heart failure symptoms. Medical management may be considered in asymptomatic patients with a small defect and a Qp: Qs of less than 1.5 [8]. Symptomatic VSDs, those measuring 10 mm or more, with a higher Qp: Qs or causing complications such as pulmonary hypertension or valvular involvement are generally closed to prevent the progression of heart failure. Management of traumatic VSDs has traditionally been surgical, which is the first choice in hemodynamically unstable patients and those with large defects not amendable to repair with a septal closure device [9, 10]. On the contrary, stable patients with smaller defects and a location with low risk of subvalvular tissue damage may undergo percutaneous management [10], which has the same success rate in the closure of VSDs as surgical repair with less complications and shorter hospital stays [11]; and has been reported to be successful in several cases of traumatic VSD [10, 12]. Our patient was stable, symptomatic, with a large defect and Qp: Qs greater than 1.5, and percutaneous treatment was successful.

Timing for closure of a traumatic VSD is controversial. In post-infarction VSD several studies have reported lower mortality rates in patients who underwent delayed surgical repair of the defect weeks after the infarction [13, 14], , allowing for fibrosis of the friable and soft infarcted tissue [3]. On the other hand, in traumatic VSD, especially in stab wounds, surrounding myocardial tissue is unscathed and early intervention after stabilization of the patient is generally reported to have favorable results [15]. In percutaneous closure of ischemic defects, due to the mentioned features of the tissue, device oversizing has been reported as a strategy to compensate for progressive tissue necrosis [16]. As in our case the etiology was traumatic, we followed the standard sizing recommendations for the selected device.

Initial myocardial injury requiring surgical repair with posterior diagnosis of a VSD case has been previously reported [3, 17], often presenting with unspecific manifestations such as persistent dyspnea and tachycardia as did our patient. Follow-up of patients with cardiac trauma has not been standardized and plays an important role in the prompt detection of complications with significant clinical implications like the presented case.

In our case, a thorough clinical evaluation and echocardiographic assessment proved to be essential for the diagnosis of a traumatic VSD. Its percutaneous management was successful and presents a viable alternative to surgical repair. A standardized follow-up of cardiac trauma patients allows for early detection of complications. Clinical suspicion, a multidisciplinary assessment, early intervention and follow-up of VSD are crucial for the success of the treatment and patient recovery.

## Electronic supplementary material

Below is the link to the electronic supplementary material.

Supplementary Material 1: Video file corresponding to Fig. : 1Video 1. Transthoracic echocardiogram. (A) Parasternal long-axis view: 13 mm ventricular septal defect (VSD). (B) Color Doppler: Left-to-right shunt across the VSD (C) Parasternal short-axis window: VSD (D) Color Doppler showing flow through the VSD

Supplementary Material 2: Video file corresponding to Fig. 3: Video 2. Transthoracic echocardiogram. Parasternal short-axis view (A) A well-positioned VSD closure device is observed in the middle segment of the anterior septum. (B) Color Doppler: Unidirectional left-to-right residual shunt, with a large decrease in flow compared to the preprocedural study

## Data Availability

No datasets were generated or analysed during the current study.

## Abbreviations

AL2: Amplatz Left 2 (catheter type)
BMW: Balanced Middle Weight (guidewire type)
CT: Computed Tomography
eFAST: Extended Focused Assessment with Sonography for Trauma
Qp: Qs: Pulmonary-to-Systemic Flow Ratio
TEE: Transesophageal Echocardiography
TTE: Transthoracic Echocardiogram
VSD: Ventricular Septal Defect
